# Pre-operative vs. post-operative stereotactic radiosurgery for operative metastatic brain tumors: study protocol for a phase III clinical trial

**DOI:** 10.1186/s12885-024-12060-9

**Published:** 2024-03-12

**Authors:** David M. Routman, Ignacio Jusue-Torres, Paul D. Brown, Daniel M. Trifiletti, Sujay A. Vora, Desmond A. Brown, Ian F. Parney, Terry C. Burns, Elizabeth Yan

**Affiliations:** 1 Department of Radiation Oncology, Mayo Clinic, 200 First Street SW, Rochester, MN 55905 USA; 2 Department of Neurological Surgery, Mayo Clinic, Rochester, MN USA; 3 Department of Radiation Oncology, Mayo Clinic, Jacksonville, FL USA; 4 Department of Radiation Oncology, Mayo Clinic, Phoenix, AZ USA; 5 Neurosurgical Oncology Unit, National Institute of Health, Bethesda, MN USA

**Keywords:** Neoadjuvant radiation therapy, Adjuvant radiation therapy, Preoperative, Postoperative, Stereotactic radiosurgery

## Abstract

**Background and Objectives:**

Almost one third of cancer patients in the United States will develop brain metastases on an annual basis. Surgical resection is indicated in the setting of brain metastases for reasons, such as maximizing local control in select patients, decompression of mass effect, and/or tissue diagnosis. The current standard of care following resection of a brain metastasis has shifted from whole brain radiation therapy to post-operative stereotactic radiosurgery (SRS). However, there is a significant rate of local recurrence within one year of postoperative SRS. Emerging retrospective and prospective data suggest pre-operative SRS is a safe and potentially effective treatment paradigm for surgical brain metastases. This trial intends to determine, for patients with an indication for resection of a brain metastasis, whether there is an increase in the time to a composite endpoint of adverse outcomes; including the first occurrence of either: local recurrence, leptomeningeal disease, or symptomatic radiation brain necrosis - in patients who receive pre-operative SRS as compared to patients who receive post-operative SRS.

**Methods:**

This randomized phase III clinical trial compares pre-operative with post-operative SRS for brain metastases. A dynamic random allocation procedure will allocate an equal number of patients to each arm: pre-operative SRS followed by surgery or surgery followed by post-operative SRS.

**Expected outcomes:**

If pre-operative SRS improves outcomes relative to post-operative SRS, this will establish pre-operative SRS as superior. If post-operative SRS proves superior to pre-operative SRS, it will remain a standard of care and halt the increasing utilization of pre-operative SRS. If there is no difference in pre- versus post-operative SRS, then pre-operative SRS may still be preferred, given patient convenience and the potential for a condensed timeline.

**Discussion:**

Emerging retrospective and prospective data have demonstrated some benefits of pre-op SRS vs. post-op SRS. This study will show whether there is an increase in the time to the composite endpoint. Additionally, the study will compare overall survival; patient-reported outcomes; morbidity; completion of planned therapies; time to systemic therapy; time to regional progression; time to CNS progression; time to subsequent treatment; rate of radiation necrosis; rate of local recurrence; and rate of leptomeningeal disease.

**Trial registration number:**

NCT03750227 (Registration date: 21/11/2018).

## General information

**Protocol title:** Pre-operative vs. Post-operative Stereotactic Radiosurgery for Operative Metastatic Brain Tumors.

**Registry:** NCT03750227; NCI-2018–02799; Clinical Trial Reporting Program (CTRP) 17–007708.

**Registration date:** 21/11/2018.

**Study dates:** November 2018 to present.

**Sponsor/Funding Agency:** Mayo Clinic Comprehensive Cancer center (NIH NCI P30CA015083); NINDS Intramural Research Program.

**Institutional approvals:** Institutional Review Board for Human Subjects Research MC167C.

**Investigator address:** 200 First Street SW. Rochester, MN 55905.

## Rationale and background information

Approximately 175,000 patients in the United States yearly, or 10–30% of cancer patients, develop brain metastases [1–5]. The current standard of care for brain metastasis is post-operative stereotactic radiosurgery (SRS) [6–12]. However, 28% of patients undergoing post-operative SRS after resectioning of one, two, or three metastases recurred locally within one year [10]. Opportunities for improvement in local control and clinical outcomes therefore remain. Dose escalation may improve local control but at the expense of toxicity including radiation-induced necrosis. Within the field of radiation oncology, there has been an increase in the utilization of neoadjuvant therapy, including but not limited to rectal, pancreatic, and esophageal cancers as well as sarcoma [13]. This strategy of neoadjuvant radiation has been gaining interest in the setting of brain metastases. Neoadjuvant SRS prior to surgical resection of brain metastases has a number of potential advantages over adjuvant SRS, including 1) a better-defined target volume delineation as opposed to a post-operative bed; 2) the potential for a “sterilization” effect prior to surgery, and therefore, the potential to decrease the rate of ‘seeding’ of viable tumor cells that could contribute to post-operative regional or leptomeningeal disease; 3) rates of radiation necrosis may be decreased with pre-operative SRS, given the potential to resect irradiated tissue surrounding the resected metastasis involving non-eloquent regions. Disadvantages include 1) the lack of pathologic confirmation prior to SRS: 2) being non-compatible with emergent surgical indications (uncommon), the potential need to decrease margin dose for large as-yet unresected lesions, and 3) the potential risk of wound healing complications operating after recent radiation [13]. Emerging retrospective and prospective data have demonstrated that some of the theoretical benefits may hold true [14–18]. Pre-operative SRS appears to be a safe and potentially effective treatment paradigm for surgical brain metastases. We present our ongoing randomized phase III clinical trial protocol comparing pre-operative with post-operative SRS for brain metastases.

## Study goals and objectives

### Primary goals

To determine for patients with brain metastases meeting the inclusion criteria, whether there is an increase in the time to a composite endpoint of adverse outcomes, including the first occurrence of either: local recurrence, leptomeningeal disease, or symptomatic radiation brain necrosis; in patients who receive SRS prior to surgery as compared to patients who receive surgery prior to SRS.

Radionecrosis will be defined radiologically based on MRI. Dynamic susceptibility weighted (DSC) imaging is preferred at each followup. Radionecrosis will be determined qualitatively by radiology per standard institutional clinical assessment. General guidelines are consistent with Alliance A221208 and other institutional trials. When radionecrosis is questionable, or uncertainty is documented, blinded central review will be performed. Relative cerebral blood volume (rCBV) in the enhancing lesion, relative to normal appearing white matter may be used, with a ratio of < 1.5 suggesting necrosis. For conventional MR, a lesion quotient, defined as the T2-weighted hypointense lesion over the maximum axial cross-sectional area of the contrast-enhancing lesion defined on the T1-weighted post-gadolinium sequence on a comparable axial slice can be considered in assessment, and rating of necrosis with a suggested quotient of < 0.3. This metric has accuracy limitations and may be more useful to rule in recurrent tumor than to rule out recurrent tumor.

Leptomeningeal disease (LMD) will be determined based on CSF cytology when available as obtained per clinical indication. Positive CSF will be classified as diffuse LMD. LMD will otherwise represent a radiologic diagnosis, per radiology, per standard institutional assessment and care. Features as below will be considered: Leptomeningeal enhancement including nodules, diffuse enhancement, and including distant sites as well as spine. Spinal imaging will be performed as clinically indicated and lumbar puncture as above will be obtained per clinical care and institutional standard. LMD will be further classified as local versus diffuse. Local: within 3 cm of the surgical bed in the absence of diffuse. LMD Diffuse: Any LMD away from surgical bed (> 3 cm) and/or positive CSF.

### Secondary goals

1. Overall Survival: to determine for patients with brain metastases whether there is improved overall survival for patients who receive SRS prior to surgery compared to patients who receive SRS after surgery.
2. Patient-Reported Outcomes: to determine for patients with brain metastases whether there is improved patient-reported outcomes, including quality of life for patients who receive SRS prior to surgery compared to patients who receive SRS after surgery.
3. Neurosurgical Morbidity: to determine if preoperative SRS increases surgical morbidity rates including postoperative complications such as wound infection, need for longer hospital stays, or readmission compared to a surgery-first approach for resectable brain metastases.
4. Completion of Planned Therapies: to determine for patients with brain metastases whether there is a higher completion rate of planned therapies for patients who receive SRS prior to surgery than patients who receive surgery prior to SRS.
5. Time to Systemic Therapy: to determine for patients with brain metastases whether there is a shorter time to initiation or re-initiation of systemic therapy with pre-operative versus post-operative SRS.
6. Time to Regional Progression, CNS Progression, and time to subsequent treatment, including WBRT: to determine for patients treated with pre-operative SRS whether there is a longer interval to regional progression, any CNS progression, or need for subsequent intracranial treatment compared to patients receiving post-operative SRS.
7. Rate of Radiation Necrosis: to determine for patients with pre-operative as compared to post-operative radiation whether there is a decreased rate of radiation necrosis, including asymptomatic and symptomatic radiation necrosis.
8. Rate of Local Recurrence: to determine for patients with pre-operative as compared to post-operative radiation whether there is a decreased rate of local recurrence.
9. Rate of Leptomeningeal Disease: to determine for patients with pre-operative as compared to post-operative radiation whether there is a decreased rate of leptomeningeal disease.

### Correlative research

1. To determine the genetic and molecular alterations of brain metastases seen after radiation versus in the resection setting alone, including early radiobiologic changes in tissue treated with SRS 24 to 48 h prior, and to investigate the detection rate of corresponding circulating DNA and/or inflammatory markers in peripheral specimens.
2. To investigate the usefulness of biomarkers and response to radiation in predicting local control and outcomes.

### Study design

This multicentric randomized phase III trial for patients with one to 10 brain metastases.

#### Study populations

The study population inclusion and exclusion criteria are described in Table 1.

**Table 1:** Study Inclusion/Exclusion criteria

**Inclusion criteria**
Age ≥ 18 years
Histological or cytological confirmation of solid tumor malignancy and/or clinical history of known or suspected metastatic disease with an intraparenchymal brain tumor consistent with brain metastasis based on clinical and radiologic findings
Clinical indication for surgical resection of one brain metastasis based on neurosurgery recommendation and patient deemed a surgical candidate
Clinical indication and plan for stereotactic radiosurgery to all known brain lesions requiring treatment (< 10 metastases)
ECOG Performance Status ≤ 2
Provide written informed consent or have a Legally Authorized Representative who is responsible for the care and well-being of the potential study participant, provide consent
Willing to return to enrolling institution for follow-up (during the Active Monitoring Phase of the study) or agreement to complete pre-specified MRI series and follow-up visits according to the study timeline (see Methodology section) mailing in digital copies of images as well as clinical notes
**Exclusion criteria**
Pregnant women
Nursing women
Men or women of childbearing potential who are unwilling to employ adequate contraception
Co-morbid systemic illnesses or other severe concurrent disease which, in the judgment of the investigator, would make the patient inappropriate for entry into this study or interfere significantly with the proper assessment of safety and toxicity of the prescribed regimens
Immunocompromised patients and patients known to be HIV positive and currently receiving antiretroviral therapy. NOTE: Patients known to be HIV positive, but without clinical evidence of an immunocompromised state, are eligible for this trial
Uncontrolled intercurrent illness including, but not limited to, ongoing or active infection, symptomatic congestive heart failure, unstable angina pectoris, cardiac arrhythmia, or psychiatric illness/social situations that would limit compliance with study requirements
Prior open neurosurgery for malignancy
Known or clinically suspected primary germ cell tumor, small cell carcinoma, or lymphoma
History of Whole Brain Radiation Therapy (WBRT)
Known allergy to gadolinium, pacemaker, or other contraindication such as metal implant that is not safe for MRI. Patients with MRI-compatible implants including MRI compatible pacemakers are eligible
Leptomeningeal metastasis/disease
A brain metastasis that is located ≤ 5 mm of the optic chiasm
Any brain metastasis > 5 cm in size
> 10 brain metastases
Indication for surgical resection of ≥ 2 brain metastases
Indication for long-term (anticipated greater than 4 weeks) 4 mg dexamethasone equivalent of steroids or bevacizumab

## Methodology

Upon enrolment, patients will be randomly assigned to one of the two arms: Arm A – pre-operative SRS followed by surgery or Arm B – surgery followed by post-operative SRS. A dynamic allocation procedure will allocate an equal number of patients to each arm. This procedure will balance the marginal distributions of the stratification factors between arms. Stratification factors include:

- Age: less than 60 versus those 60 or older
- Number of brain metastases: 1 vs. 2–4 vs. 5–10
- Tumor Size: Planned resection of metastasis < 3.0 cm vs. > 3.0 cm (but < 5.0 cm)
- Primary Malignancy: Lung vs. Radioresistant (melanoma, renal cell carcinoma, sarcoma) vs. Other
- Dural Contact: Yes, versus No, with dural contact defined radiologically as suspicion of loss of a plane between the tumor and dura or within 1 mm.
- RT modality: SRS planned as LINAC vs. Gamma knife

### Randomization

**Fig. 1:**
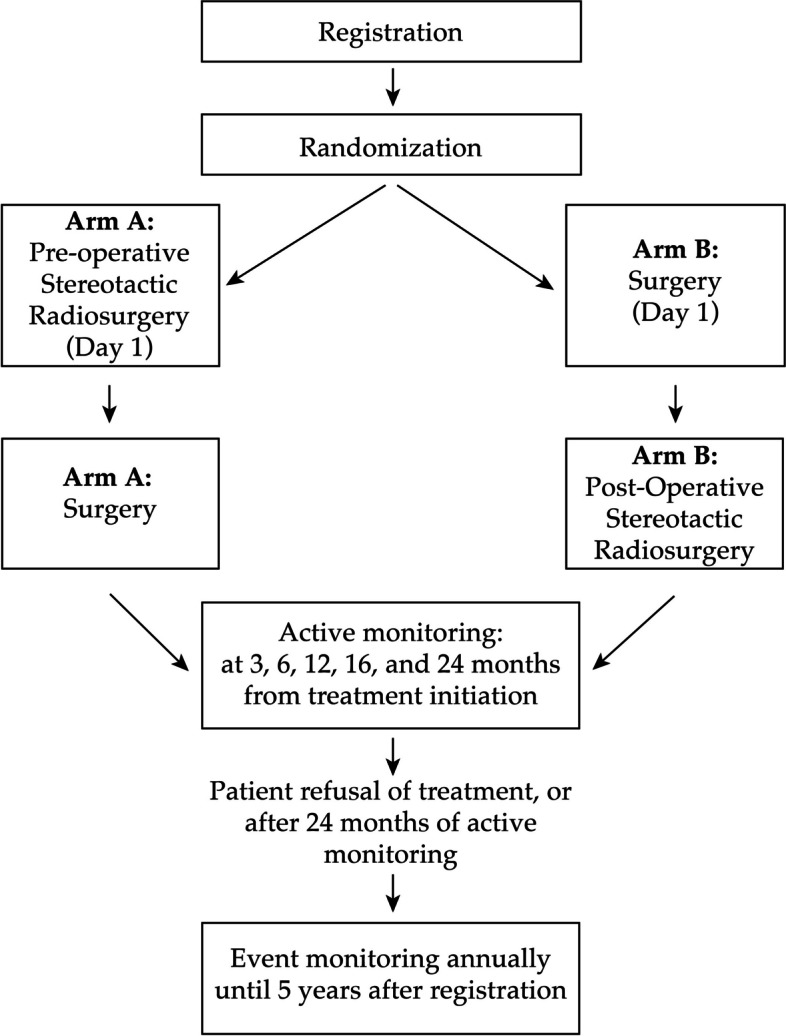
Study time flow diagram from registration, to randomization, to intervention, to followup

The balancing algorithm that we will use is a dynamic allocation procedure that is part of iMedidata Rave, known as Balance. After stratification (Fig. 1), patients will be randomized at a 1:1 ratio between:

- **Arm A:** Pre-op SRS followed by surgery (within two weeks post-SRS).
- **Arm B:** Surgery followed by post-op SRS (within four weeks of surgery).

### Radiation fractionation and target dose

The dose prescription is to be delivered in a single SRS fraction. The volume of the metastasis will determine the total prescribed dose. For pre-operative SRS, the contrast enhanced tumor planned for resection is the clinical gross tumor volume (CGTV), and defines the clinical target volume (with an optional 1 mm expansion for dural contact)  (GTV = CTV) (Arm A). For post-operative SRS,  the surgical bed with a 1 mm margin will be used as the CTV (Arm B). 

For GKRS, the dose should be prescribed to the highest isodose line encompassing the clinical target volume (CTV), which can range from 50 to 90% of the maximum dose.

For LINAC-based SRS, the target volume should be prescribed to a planning target volume (PTV) representing a 1 to 2 mm expansion on the CTV. The maximum dose to the target defined as dmax should be a minimum of 125%.

### Radiation therapy dosing:

- Lesions < 4.2 ccs receive 20 Gy (22 Gy is allowed for subcentimeter metastases)
- Lesions ≥ 4.2c to <8.0 cc receive 18Gy
- Lesions ≥ 8.0 to <14.4 cc receive 17Gy
- Lesions ≥ 14.4 to <20cc receive 15Gy
- Lesions ≥ 20 to <30cc receive 14Gy
- Lesions ≥ 30cc max 12Gy

### Sample size and power

Statistical considerations regarding sample size and study power are based on the primary endpoint CNS composite endpoint event (CNS-CE). In Mayo Clinic’s experience, the time to CNS-CE for the Sx-SRS group is about six months. Using the log-rank test with a one-sided alpha level of 0.10 a total of 116 evaluable patients (58 in Group A and 58 in Group B) will provide 90% power to detect at least a four-month increase in median time to CNS-CE event from 6.0 months to 10.0 month in Arm B (post-operative SRS) versus Arm A (pre-operative SRS) (EAST 6.4). The final analysis will be conducted when 104 events have occurred; or when all patients are either censored or have been followed for at least 24 months, whichever occurs first. We anticipate accruing an additional 24 patients to account for ineligibility, non-evaluability, and cancellations. This design has a 46.2% chance of stopping early if there is no difference between the treatment groups and only a 2.5% chance if the Sx-SRS group has a greater time to CNS-CE event time than the SRS-Sx group. Note: In this patient population, the expected drop-out rate due to lost-to-follow-up is less than 2%; therefore, most patients are expected to be evaluable for the primary and secondary endpoints.

## Discussion

Emerging retrospective and prospective data have demonstrated some of the theoretical benefits of preop SRS vs. postop SRS [14–18]. At the time of this publication, we are aware of two ongoing Phase 2 clinical trials [19, 20] and five additional ongoing Phase 3 randomized clinical trials actively enrolling patients NCT03741673 (MD Anderson, enrolment target = 110), NCT04474925 (Alberta, enrolment target = 88), NCT05124236 (Multicentric International Europe, enrolment target = 200), NCT05438212 (NRG Oncology NCI, enrolment target = 236), NCT05545007 (IRCCS Istituto Clinico Humanitas, Milano, Italy, enrolment target = 170). The current study NCT03750227 has an enrolment target of 140 patients and will provide information on whether there is an increase in the time to a composite endpoint of occurrence of either: local recurrence, leptomeningeal disease, or symptomatic radiation brain necrosis in patients who receive SRS prior to surgery as compared to patients who receive surgery prior to SRS. Additionally, the study will compare overall survival; patient-reported outcomes; morbidity; completion of planned therapies; time to systemic therapy; time to regional progression; time to CNS progression; time to subsequent treatment; rate of radiation necrosis; rate of local recurrence; and rate of leptomeningeal disease for pre-op vs. post-op SRS.

As designed, our study design has multiple limitations, some of which are addressed in other ongoing studies: Primary among these is the inflexibility of the radiation dose and fractionation. Dosing for MC167C builds upon the experience of NCCTG N107C/CEC·3 [9], which compared post-operative SRS to post-operative WBRT. This dosing paradigm means that some of the largest eligible lesions may be treated with as little as 12 Gy radiation, potentially creating hesitation to enroll patients with large lesions on this protocol. This challenge could be addressed via dose escalation especially in pre-operative radiation; wherein irradiated tissue, including some surrounding brain tissue, is intentionally resected when safely achievable but is not permitted on our protocol. Alternatively, fractionation may enable larger lesions to be safely treated to more biologically effective radiation doses. The ongoing FRACTIONATE study (NCT05222620) directly compares single fraction versus fractionated radiosurgery to treat surgical cavities; fractionated radiation is utilized in the post-operative arm for NCT05124236 and in both the pre-and post-operative arms for NCT05545007.

We are heartened by the strong interest in evaluating neoadjuvant SRS, as evidenced by the multiple active trials addressing this important question. We look forward to managing patients in the future based on the results of these rigorously designed randomized studies.

### Trial status

At the time of manuscript submission, the trial status is recruiting. Sixty-five patients have been enrolled. Criteria for interim analysis still need to be met.

### Safety considerations

Depending on random study selection, the Cancer Center Auditing area will review the trial bi-annually or yearly to assess accrual, adverse events, and any endpoint problems. Any safety issues requiring protocol changes will be communicated through protocol amendments.

This study will be reviewed in conjunction with the Mayo Clinic Cancer Center Data and Safety Monitoring Board (DSMB) processes as an interventional study. Any safety issues requiring protocol changes will be communicated through protocol amendments.

The principal investigator and the statistician will review the study at least twice a year to identify accrual, adverse events, and any endpoint problems that might develop.

### Follow-up

All enrolled patients will be followed up for a total of 5 years. For the first two years, post treatment patients will receive active monitoring every three months, undergoing history, physical and neurological exam, including weight, medication review and ECOG performance status, MRI, and adverse events assessment. After two years, patients will be monitored for key study events such as progression, new primaries, and death up to 5 years post enrolment.

### Data management and statistical analysis

All data will be entered into electronic case report forms (eCRF’s) through the Medidata Rave system. Case report forms will be automatically rolled out based on a predetermined and visit-based schedule to improve study staff workflow and data quality. Data will be exported nightly to a secure FTP for analysis and reporting.

The main objective is to assess whether patients with brain metastases who meet the eligibility criteria experience a longer time until a combined outcome of negative events, such as local recurrence, leptomeningeal disease, or symptomatic radiation brain necrosis, when they undergo SRS before surgery compared to those who undergo surgery before SRS.

Efficacy analysis will be based on the intention-to-treat principle with all eligible patients belonging to the treatment group to which they were randomized. A sensitivity analysis will also be performed based on the treatments received, comparing pre-operative SRS to post-operative SRS. Based on cavity size, a sensitivity analysis will also be performed for patients assigned to post-operative SRS who cannot receive post-operative SRS.

Interim Analysis: One interim futility analysis will be conducted when 50% (52 events) of the CNS-CE events have occurred (approximately 35 months after the study opens). If the hazard ratio comparing Arm A (pre-operative SRS) to Arm B (post-operative SRS is greater than 1.032, we will conclude that the SRS-Sx (pre-operative) treatment is not superior to Sx-SRS (post-operative) and report that the result; otherwise, conclusions will wait until the end of the study.

### Quality assurance

Each eCRF will contain edit checks and custom functions to ensure the highest possible data quality. Only necessary eCRFs will be available for data entry to reduce the possibility of erroneous entries. The edit checks and custom functions on the eCRFs will trigger queries requesting the attention of appropriate study staff. The fields will be marked in pink to allow study staff to identify the data fields that require attention or actions quickly. Additionally, secure email notifications will be sent for adverse event tracking and monitoring.

### Expected outcomes of the study

Our trial could substantially influence practice and patient care if composite clinical endpoints are better with pre-compared to post-operative SRS. If pre-operative SRS improves outcomes relative to post-operative SRS, this will establish pre-operative SRS as superior. If there were a negative result (understanding this is not a non-inferiority trial), it would lend more support to current clinical practices. If there is no difference in pre- versus post-operative SRS, then pre-operative SRS may still be preferred, given patient convenience and the potential for a condensed timeline. If post-operative SRS proves superior to pre-operative SRS, it will remain a standard of care and halt the increasing utilization of pre-operative SRS.

### Duration of the project

The estimated accrual period will be 4.5 years, and the total study duration will be 6.5 years. One hundred forty patients are expected to be accrued, including an extra 24 to accommodate losses due to cancellations, ineligibility, non-evaluability, or major protocol violations. The total number of evaluable patients needed is 116 (58 per arm). Based on Mayo Clinic experience, the accrual rate for this trial will be approximately 31 eligible patients per year (about 2.5 per month). The final statistical analysis will conclude within 12 months of trial completion.

### Project management

The study is being led by the principal investigator Dr. Elizabeth Yan. She will oversee study investigators in all aspects of the study conduct. Data interpretation and dissemination of results will be managed under her direct supervision.

## Data Availability

The datasets generated and/or analyzed during the current study are not publicly available because the current publication is the study protocol of an ongoing clinical trial, which still enrolls patients but is available from the corresponding author upon reasonable request.

## Abbreviations

AE: Adverse event/adverse experience
CFR: Code of federal regulations
CNS: Central nervous system
CRF: Case report form
CTV: Clinical target volume
DICOM: Digital imaging and communication
DSMB: Data and safety monitoring board
FDA: Food and drug administration
GCP: Good clinical practice
GTV: Gross tumor volume
GKRS: Gamma knife radiosurgery
Gy: Gray (radiation dosing)
HIPAA: Health insurance portability and accountability act
IRB: Institutional review board
LINAC: Linear accellerator
LMD: Leptomeningeal disease
MRI: Magnetic resonance imaging
NCI: National cancer institute
NINDS: National institute of neurologic disorders and stroke
NRG Oncology: NSABP, RTOG, GOG. It is a National Clinical Trials Network (NCTN) group involving the National Surgical Adjuvant Breast and Bowel Project (NSABP), the Radiation Therapy Oncology Group (RTOG), and the Gynecologic Oncology Group (GOG)
NTD: Normal tissue dose
OS: Overall survival
PHI: Protected health information
PI: Principal investigator
PS: Performance score or performance status
PTRAX: Research participant tracking system
PTV: Planning target volume
QOL: Quality of life
RT: Radiation therapy
RTOG: Radiation therapy oncology group
SAE: Serious adverse event/serious adverse experience
SOP: Standard operating procedure
SRS: Stereotactic radiosurgery
UPIRTSO: Unanticipated problems involving risks to subjects or others
WBRT: Whole brain radiation therapy
